# Genomic African and Native American Ancestry and Chagas Disease: The Bambui (Brazil) Epigen Cohort Study of Aging

**DOI:** 10.1371/journal.pntd.0004724

**Published:** 2016-05-16

**Authors:** M. Fernanda Lima-Costa, James Macinko, Juliana Vaz de Mello Mambrini, Sérgio Viana Peixoto, Alexandre Costa Pereira, Eduardo Tarazona-Santos, Antonio Luiz Pinho Ribeiro

**Affiliations:** 1 Fundação Oswaldo Cruz, Instituto de Pesquisas René Rachou, Belo Horizonte, Brazil; 2 University of California, Fielding School of Public Health, Departments of Health Policy and Management and Community Health Sciences, Los Angeles, California, United States of America; 3 Universidade Federal de Minas Gerais, Escola de Enfermagem, Departamento de Enfermagem Aplicada, Belo Horizonte, Brazil; 4 Universidade de São Paulo, Instituto do Coração, São Paulo, Brazil; 5 Universidade Federal de Minas Gerais, Instituto de Ciências Biológicas, Departamento de Biologia Geral, Belo Horizonte, Brazil; 6 Universidade Federal de Minas Gerais, Hospital das Clínicas e Faculdade de Medicina, Belo Horizonte, Brazil; New York University, UNITED STATES

## Abstract

**Background:**

The influence of genetic ancestry on *Trypanosoma cruzi* infection and Chagas disease outcomes is unknown.

**Methodology/Principal Findings:**

We used 370,539 Single Nucleotide Polymorphisms (SNPs) to examine the association between individual proportions of African, European and Native American genomic ancestry with *T*. *cruzi* infection and related outcomes in 1,341 participants (aged ≥ 60 years) of the Bambui (Brazil) population-based cohort study of aging. Potential confounding variables included sociodemographic characteristics and an array of health measures. The prevalence of *T*. *cruzi* infection was 37.5% and 56.3% of those infected had a major ECG abnormality. Baseline *T*. *cruzi* infection was correlated with higher levels of African and Native American ancestry, which in turn were strongly associated with poor socioeconomic circumstances. Cardiomyopathy in infected persons was not significantly associated with African or Native American ancestry levels. Infected persons with a major ECG abnormality were at increased risk of 15-year mortality relative to their counterparts with no such abnormalities (adjusted hazard ratio = 1.80; 95% 1.41, 2.32). African and Native American ancestry levels had no significant effect modifying this association.

**Conclusions/Significance:**

Our findings indicate that African and Native American ancestry have no influence on the presence of major ECG abnormalities and had no influence on the ability of an ECG abnormality to predict mortality in older people infected with *T*. *cruzi*. In contrast, our results revealed a strong and independent association between prevalent *T*. *cruzi* infection and higher levels of African and Native American ancestry. Whether this association is a consequence of genetic background or differential exposure to infection remains to be determined.

## Author Summary

Chagas disease (ChD), which is caused by the protozoan *Trypanosoma cruzi*, affects approximately 8 million people worldwide. ChD is known as a neglected tropical disease. The disease is endemic in South and Central American countries, and is an emerging issue in North America and Europe. This study examined, for the first time, the association between genomic ancestry and *T*. *cruzi* infection, Chagasic cardiomyopathy and its ability to predict long term mortality. Our results show that persons with higher levels of African and Native American ancestries (and the reverse for European ancestry) are more likely to be infected with *T*. *cruzi*. However, genomic ancestry had no effect on either Chagasic cardiomyopathy or on its ability to predict mortality. Whether the association between *T*. *cruzi* infection and genomic ancestry is a consequence of genetic susceptibility or differential exposure to infection due to poor socioeconomic circumstances over the life course, remains to be determined.

## Introduction

Chagas disease (ChD), which is caused by the protozoan *Trypanosoma cruzi*, affects approximately 5.7 million people in 21 Latin American countries [1]. ChD is known as a neglected tropical disease and is an emerging issue in North America and Europe [2–5]. ChD is autochthonous in South and Central America but *T*. *cruzi* infection has spread to other regions of the world primarily due to immigration of infected persons [2], although there has been evidence of some locally-occurring infections in the United States [3]. Currently, at least 300,000 persons with *T*. *cruzi* infection live in the US [4] and at least 80,000 in Europe [5]. The disease is costly to individuals and society with estimates of over USD 100 million spent on treatments and over USD 800 million in lost productivity each year [6]. Up to one third of those infected with ChD may develop chronic heart abnormalities and other complications of which Chagas cardiomyopathy is the most severe and life-threatening form [7]. The presence of major electrocardiogram (ECG) abnormalities (a diagnostic measure of Chagas cardiomyopathy) doubles the risk for mortality in *T*. *cruzi*-infected elderly populations [8].

The influence of African and/or Native American ancestry on *T*. *cruzi* infection and/or ChD outcomes is unknown. The existence of an association is plausible for at least two reasons: first, familial aggregation of *T*. *cruzi* seropositivity and ECG abnormalities have been found in highly endemic areas, suggesting that genetic variation may play a role in susceptibility to infection as well as disease progression [9,10]; second, an earlier publication, using ethnoracial self-classification, reported greater prevalence of ECG abnormalities among Black middle-aged adults relative to their White counterparts [11].

Latin America is one of the most ethnoracially heterogeneous regions of the world [12], and Brazil is the largest and the most populous ChD endemic country in the region. The current Brazilian population’s genetic makeup is the product of admixture between Amerindians, Europeans colonizers or immigrants, and African slaves [13]. Brazil received nearly 4 million slaves from Africa, about seven times more than the United States [14]. Thus, the Brazilian population provides an opportunity to assess the relationship between *T*. *cruzi* infection and its complications with genetic ancestry in admixed populations.

The Bambui-Epigen Cohort Study of Aging is conducted in a well-defined population of older Brazilian adults living in a formerly ChD endemic area [15]. We examined for the first time the association between genome-wide proportions of genomic ancestry with *T*. *cruzi* infection and cardiomyopathy, taking into account an array of socioeconomic and health indicators that could confound such an association. Additionally, we examined whether genomic ancestry affects the prognostic value of major ECG abnormalities for 15-year mortality in *T*. *cruzi*-infected individuals.

## Methods

### Study design and population

The Bambui cohort study of aging is ongoing in Bambuí, a city of approximately 15,000 inhabitants in the state of Minas Gerais in Southeast Brazil, which is one of the oldest known endemic areas for ChD [16–18]. Detailed information on this cohort can be found elsewhere [15]. Briefly, the population eligible for the cohort consisted of all residents aged 60 years and over on 1 January 1997 (92% of the 1,742 inhabitants in this age group participated). Most participants had some degree of admixture between African, European and Native American genomic ancestry [19,20].

### *T*. *cruzi* infection

*T*. *cruzi* infection status was assessed by means of three different assays performed concurrently: a hemagglutination assay (Biolab Merieux SA, Rio de Janeiro, Brazil) and two enzyme-linked immunosorbent assays (Abbott Laboratories, Inc., North Chicago, Illinois; and Wiener Laboratories, Rosario, Argentina). Infection with *T*. *cruzi* was defined by seropositivity in all of the three examinations; seventeen persons had discordant results among the assays and were excluded from the analysis. As far as we could determine, none of the cohort participants had a history of use of antitrypanosomal medications, and none of the seropositive subjects reported such treatment over the ensuing decade during annual follow-up visits. Thus, the use of antitrypanosomal therapy was not considered in the present analysis. In addition, no cohort participant had received a cardiac transplant.

### Electrocardiogram

At the baseline examination, a digitally recorded 12-lead ECG (Hewlett Packard MI700A) reading was obtained at rest. ECGs were analyzed at the ECG Reading Center (EPICARE, Wake Forest University) and classified using the Minnesota Code (MC) criteria [21,22]. Major ECG abnormalities were defined by the presence of at least one of the following: old (MC 1.1.x or 1.2.x) or possible myocardial infarction (1.3.x and 4.1.x, 4.2, 5.1, or 5.2), complete intraventricular blocks (MC 7.1, 7.2, 7.4, or 7.8), frequent supraventricular or ventricular premature beats (MC 8.1.x, except 8.1.4), major isolated ST segment or T-wave abnormalities (MC 4.1.x, 4.2, 5.1 or 5.2), atrial fibrillation or flutter or supraventricular tachycardia (MC 8.3.x.or 8.4.2), other major arrhythmias (MC 8.2.x, except 8.2.1), major atrioventricular conduction abnormalities or pacemaker use (MC 6.1, 6.2.x, 6.4, 6.8, 8.6.1 or 8.6.2), major QTi prolongation (>115%) and left ventricular hypertrophy (LVH) (MC 3.1 together with [4.1.x, 4.2, 5.1, or 5.2]). Further details can be seen elsewhere [8].

### Genetic and ancestry analyses

Cohort participants were genotyped with the Omni 2.5M array (Illumina, San Diego, California) [13]. We performed ancestry inferences using the model-based method [23], implemented in the Admixture software. First, we used 370,539 SNPs to estimate for each individual African, European and Native American tri-hybrid ancestry proportions, using 266 African, 262 European and 93 Native American individuals from public datasets as parental populations [13]. Further, we inferred a kinship coefficient for each pair of individuals, using the software Reap [24], conditioning on tri-hybrid individual admixture proportions. We used complex networks to identify families from the matrix of pair-wise kinship coefficients [13]. In this approach, pairs of individuals (i.e. families) are related if they have a kinship coefficient >0.1 (first and second-degree relatives). Given that Brazilians with African ancestry generally have a high proportion of East African genetic markers (as opposed to markers of West African origin), relative to African Americans and those from the Caribbean [13,25,26], we used 331,790 SNPs and the reference dataset “U” [13] to further divide total African ancestry into its two components: a Western-African/non Bantu and an Eastern African/Bantu, hereafter called Western African and Eastern African, respectively. The fact that many Bambuí residents are related could affect high-resolution inferences of biogeographic ancestry (such as West- and East-African) with the Admixture software. To overcome this limitation, we performed separate Admixture runs to infer West- and East- African ancestry components, avoiding the presence of related individuals in the same run. Further details on how genetic and ancestry analyses of the Bambui cohort population were performed can be found elsewhere [13,27].

### Mortality

Deaths that occurred between study enrollment in 1997 and December 31, 2011, were included in the present analysis. Deaths were reported by next of kin during the annual follow-up interview and verified through the Brazilian mortality information system. Death certificates were obtained for 95.7% of the participants who died. Deaths from any cause were considered in this analysis.

### Other variables

Potential confounding variables included baseline sociodemographic characteristics (age, sex, schooling, household income and father’s occupation) and health measures (current smoking, hypertension, diabetes, coronary heart disease, C-reactive protein and non-HDL cholesterol level). We categorized schooling into incomplete primary school (<4 years) and complete primary and higher (4 years and more). We categorized monthly household income per capita into equal or superior to the median value (median = 1.5 Brazilian minimum wages or USD 180 in 1997). Occupation of the study participant’s father (as informed by cohort members) was categorized into urban workers, landowners, manual rural workers and unknown. Current smokers were persons who had smoked at least 100 cigarettes during their lifetime and who still smoke. Body mass index (BMI) was defined as weight (in kg) divided by height (in meters) squared. Hypertension was defined by mean (two out of three measures) systolic blood pressure of ≥140 mmHg and/or diastolic pressure of ≥90 mmHg and/or treatment [28]. Diabetes mellitus was defined by fasting blood glucose ≥126mg/dL and/or treatment [29]. Coronary heart disease was defined by prior medical diagnosis of myocardial infarction and/or symptoms of angina pectoris [30]. High sensitivity C-Reactive Protein was measured by the CRP immunonephelometric method (BNII, Dade Behring, Marburg, Germany). Blood fasting glucose and cholesterol were determined by using standard enzymatic methods (Merck, Darmstadt, Germany). Non-HDL cholesterol was defined by total cholesterol level minus HDL cholesterol.

### Statistical analysis

Unadjusted analyses were based on Pearson´s chi square, oneway ANOVA and Kruskall Wallis tests to examine differences across frequencies, means and medians, respectively. Individual proportions of genomic ancestries were expressed as medians or divided into quintiles.

Prevalence ratios (PR) estimated by multivariable Poisson regression [31] were computed to examine associations between (i) genomic ancestry in quintiles and *T*. *cruzi* infection and (ii) genome ancestry in quintiles and major ECG abnormality among persons infected with *T*. *cruzi*. Further, we used Cox proportional hazard models to implement an analysis restricted to persons infected with *T*. *cruzi* to assess the influence of each category of genomic ancestry on the risk of major ECG abnormalities and subsequent mortality.

The above-mentioned statistical analyses were based on two models. First, prevalence and hazard ratios were adjusted for age (continuous), sex, smoking, hypertension, diabetes, coronary heart disease (all dichotomous variables) plus body mass index, log-transformed C-reactive protein and non-HDL cholesterol (as continuous measures). We then added schooling, monthly household income per capita, and father’s occupation to the previous models. Because 913 participants were first- or second-degree relatives, and excluding them would lead to loss of power and possible selection bias, we kept all related individuals in our analyses and used robust variance estimators in multivariate models to correct results for clustering by family structure. Finally, we examined separately the significance of the effect of multiplicative interactions between sex and genomic ancestry on each outcome by means of cross-product terms in Poisson and Cox proportional hazards regression models, respectively. Since there was no evidence of interaction with sex, the analyses were carried out for both men and women with sex included as a covariate.

Separate analyses were performed for African, Native American and European genomic ancestries and further for Western African sub continental ancestry. Statistical analyses were conducted using STATA 13.0 statistical software (Stata Corporation, College Station).

### Ethics assessment

The Bambui cohort study of aging was approved by the Institutional Review Board of the Oswaldo Cruz Foundation, Rio de Janeiro, Brazil. Genotyping was approved by Brazil’s national research ethics committee, as part of the Epigen-Brazil protocol (CONEP, resolution 15895). Written informed consent was obtained from all participants at baseline and at all follow-up interviews.

## Results

Of the 1,606 baseline cohort participants, 1,343 had complete information for all study variables and were included in the current analysis. As shown in Table 1, the prevalence of *T*. *cruzi* infection was 37.6% (n = 505). At baseline, the mean age of participants was 68.8 years, 61.2% were women, and low schooling level (<4 years) largely predominated (64.1%). The median proportions of African, Native American and European genomic ancestries were 9.6%, 5.4% and 83.8%, respectively. The median proportion of Western African sub-continental ancestry relative to total African ancestry was 63.9% (complementarily, the corresponding value for Eastern African ancestry was 36.1%). *T*. *cruzi* infected participants had significantly higher median individual proportions of African and Native American ancestries and significantly lower median European genomic ancestry. Other baseline characteristics of the study participants, by *T*. *cruzi* infection status, are presented in Table 1.

**Table 1:** Selected baseline characteristics of study participants, and by *Trypanosoma cruzi* infection (The Bambui Cohort Study of Ageing).

Characteristics	Total	Infection	
	(n = 1343)	Yes (n = 505)	No (n = 838)
Age in years, mean (SD)	68.8 (7.0)	69.2 (6.9)	68.6 (7.0)
Female sex, %	61.2	67.9	57.2^a^
< 4 years of schooling	64.1	84.8	51.7^b^
Monthly household income per capita < 180 USD	46.9	52.7	43.4^b^
**Father’s occupation, %**			
Urban	11.4	4.2	15.8^a^
Rural: landowner	25.4	16.5	30.8
Rural: manual worker	52.4	67.6	43.3
Unknown	10.7	11.7	10.1
Current smokers, %	17.7	16.8	18.3
Any major ECG abnormality, %	41.0	56.4	31.7^a^
Body mass index in kg/m^2^, mean (SD)	25.1 (4.9)	24.4 (5.1)	25.6 (4.8)^b^
Hypertension, %	61.3	60.4	61.8
Diabetes, %	14.6	10.7	17.0^b^
Coronary heart disease, %	13.1	17.6	10.4^b^
High sensitivity C-reactive protein in mg/L, median (IQR)	3.17 (4.93)	3.08 (4.92)	3.25 (4.94)
Non-HDL cholesterol in mg/dL, mean (SD)	185.0 (50.0)	184.0 (51.2)	185.6 (51.2)
African genomic ancestry, median (IQR)	9.6 (12.7)	12.8 (15.2)	8.4 (10.5)^a^
Native American genomic ancestry, median (IQR)	5.4 (5.6)	7.1 (5.9)	4.6 (4.8)^a^
European genomic ancestry, median (IQR)	83.8 (16.9)	78.6 (19.4)	86.1 (15.2)^a^
**Proportion of sub continental ancestry relative to total African genomic ancestry**			
Western African, median (IQR)	63.9 (28.9)	65.0 (23.5)	62.5 (31.9)
Eastern African, median (IQR)	36.1 (28.9)	35.1 (23.5)	37.5 (31.9)

SD, standard deviation; ECG, electrocardiogram; IQR, interquartile range; HDL, high-density lipoprotein cholesterol.

^a^p<0.001, Pearson´s chi square, one way ANOVA or Kruskall Wallis tests for differences between frequencies, means and medians, respectively.

^b^p<0.01, Pearson´s chi square, one way ANOVA or Kruskall Wallis tests for differences between frequencies, means and medians, respectively.

Table 2 presents median individual proportions of African, Native American and European genomic ancestries by baseline characteristics. Median African and Native American genomic ancestries were significantly higher (and European ancestry was significantly lower) among those with lower schooling and income levels, those whose fathers were manual workers or had an unknown occupation, as well as those with any major ECG abnormality or previous coronary heart disease. Median African ancestry was lower in those aged 69 years and over and in those with BMI under 25 kg/m^2^. No significant associations with genomic ancestry were found for other study variables.

**Table 2:** Baseline characteristics of study participants significantly associated with African, Native American and/or European genomic ancestry (The Bambui Cohort Study of Ageing).

Characteristics	African	Native American	European
	Median (IQR)	Median (IQR)	Median (IQR)
**Age in years**			
<69	10.3 (13.2)	3.5 (4.7)	83.1 (17.6)
> = 69 (above the mean)	9.0 (11.8)^a^	3.2 (4.2)	84.9 (16.8)^b^
**Schooling in years**			
> = 4	6.7 (8.7)	2.5 (3.4)	89.0 (13.0)
<4	11.5 (13.2)^b^	4.0 (5.0)^b^	80.6 (17.1)^b^
**Monthly household income per capita**			
< 180 USD (below the median)	11.0 (13.5)	3.9 (4.7)	81.5 (18.3)
> = 180 USD	8.9 (10.9)^b^	2.9 (4.2)^b^	85.3 (16.3)^b^
**Father’s occupation, %**			
Urban	7.6 (9.2)^b^	2.5 (3.8)^b^	87.7 (14.3)^b^
Rural: landowner	6.6 (8.7)	2.7 (3.4)	88.7 (12.7)
Rural: manual worker	11.5 (13.4)	4.0 (5.1)	80.7 (16.9)
Unknown	11.8 (17.4)	3.9 (5.0)	80.0 (21.7)
**Any major ECG abnormality**			
No	9.2 (11.8)	3.2 (4.5)	84.7 (16.9)
Yes	10.3 (13.3)^c^	3.5 (4.5)^c^	82.5 (17.6)^a^
**Body mass index in kg/m** ^**2**^			
< 25	10.0 (13.2)	3.5 (4.6)	83.4 (18.4)
> = 25	9.4 (11.6)^c^	3.2 (4.4)	84.4 (16.2)^c^
**Coronary heart disease**			
No	9.4 (12.3)	3.3 (4.4)	84.2 (16.7)
Yes	10.7 (13.6)^c^	3.9 (5.2)^c^	81.1 (17.6)^c^

IQR, interquartile range; ECG, electrocardiogram.

^a^p<0.01, Kruskall Wallis tests for differences between medians.

^b^p<0.001, Kruskall Wallis tests for differences between medians.

^c^p<0.05, Kruskall Wallis tests for differences between medians.

Associations between the different genomic ancestries and *T*. *cruzi* infection are shown in Table 3. There was a graded positive univariate association between *T*. *cruzi* infection with the proportion of African and Native American ancestry, and a graded negative relationship with a greater proportion of European ancestry (p<0.001 for all). After adjustments for age, sex and health measures, persons at the intermediate and highest quintiles of African and Native American ancestry were significantly more likely to be infected with *T*. *cruzi* relative to their counterparts in the lowest quintiles. After further adjustments for socioeconomic indicators (schooling, income and father’s occupation) these associations were attenuated, but remained largely significant (PR = 1.38; 95% CI 1.07, 1.79 and PR = 1.74; 95% CI 1.37, 2.35 for those at the intermediate and highest quintiles of African ancestry, respectively, and PR = 1.54; 95% CI 1.19, 1.99 for those at the highest quintile of Native American ancestry). The opposite trend was found for European ancestry (PR = 0.73; 95% CI 0.63, 0.85 and PR = 0.54; 95%CI 0.41, 0.70, respectively).

**Table 3:** Association between individual proportion of African, Native American and European genomic ancestry levels with *Trypanosoma cruzi* infection (The Bambui-Epigen Cohort Study of Aging).

Genome ancestry in quintiles	Number	% Infected with *T*. *cruzi*	PR (95% CI) adjusted for age, sex and health measures^a^	PR (95% CI) adjusted for age, sex, health measures^a^ and socioeconomic indicators^b^
**African**				
Lowest (<4.2%)	269	21.2	1.0	1.0
2nd-4th (4.2%-19.9%)	808	37.4	1.73 (1.32, 2.27)	1.38 (1.07–1,79)
Highest (≥20%)	266	54.9	2.52 (1.90, 3.35)	1.74 (1.37–2.35)
		p<0.001		
**Native American**				
Lowest (<2.3%)	272	22.8	1.0	1.0
2nd-4th (2.3%-9.3%)	807	37.3	1.63 (1.26, 2.10)	1.22 (0.96, 1.55)
Highest (≥9.4%)	264	53.8	2.37 (1.81, 3.10)	1.54 (1.19, 1.99)
		p<0.001		
**European**				
Lowest (<71%)	269	57.3	1.0	1.0
2nd-4th (71%-92.7%)	806	36.7	0.65 (0.55–0.76)	0.73 (0.63, 0.85)
Highest (≥93%)	268	20.5	0.36 (0.27–0.48)	0.54 (0.41, 0.70)
		p<0.001		

PR (95% CI), prevalence ratio (95% confidence interval) estimated by Poisson regression; p, p value—Pearson´s chi square test.

^a^Smoking, body mass index, hypertension, diabetes, coronary heart disease, log high sensitivity C-reactive protein and non-HDL cholesterol plus cluster in family.

^b^Schooling level, monthly household income per capita and father’s occupation.

Among those infected, 56.4% had at least one major ECG abnormality (31.7% among the non-infected). As shown in Table 4, in the bivariate analysis, a major ECG abnormality among infected persons was not found to be significantly (p>0.05) associated with African, Native American or European ancestry levels. This absence of association remained in analyses adjusted for age, sex and health measures, as well as in analyses further adjusted for socioeconomic indicators.

**Table 4:** Association between individual proportion of African, Native American and European genomic ancestry levels with any major electrocardiogram abnormalities among infected with *Trypanosoma cruzi* (The Bambui-Epigen Cohort Study of Aging).

Genomic ancestry in quintiles	Number	% with any major ECG abnormality	PR (95% CI) adjusted for age, sex and health measures^a^	PR (95% CI) adjusted for age, sex, health measures^a^ and socioeconomic indicators^b^
**African**				
Lowest (<4.2%)	57	57.9	1.0	1.0
2nd-4th (4.2%-19.9%)	302	57.0	1.00 (0.79,1.25)	0.99 (0.78, 1.24)
Highest (≥20%)	146	54.8	0.96 (0.73, 1.27)	0.96 (0.73, 1.26)
		p = 0.886		
**Native American**				
Lowest (<2.3%)	62	56.5	1.0	1.0
2nd-4th (2.3%-9.3%)	301	57.1	1.02 (0.71, 1.97)	1.01 (0.69, 1.48)
Highest (≥9.4%)	142	54.9	0.99 (0.66, 1.48)	0.98 (0.64, 1.49)
		p = 0.908		
**European**				
Lowest (<71%)	154	52.6	1.0	1.0
2nd-4th (71%-92.7%)	294	58.1	1.11 (0.91, 1.36)	1.11 (0.91, 1.35)
Highest (≥93%)	55	58.2	1.04 (0.77, 1.39)	1.05 (0.79, 1.40)
		p = 0.515		

ECG, electrocardiogram; PR (95% CI), prevalence ratio (95% confidence interval) estimated by Poisson regression; p, p value—Pearson´s chi square test.

^a^Smoking, body mass index, hypertension, diabetes, coronary heart disease, log high sensitivity C-reactive protein and non-HDL cholesterol plus cluster in family.

^b^Schooling level, monthly household income per capita and father’s occupation.

Over a 15 year follow-up period, 683 participants died and 109 (8.1%) were lost to follow-up, leading to 14,680 person-years (pyrs) of observations (5,251 pyrs among the infected). The death rate was 46.4 per 1,000 pyrs (56.2 and 40.9 per 1,000 pyrs among *T*. *cruzi* infected and non-infected, respectively). As shown in Table 5, persons infected with *T*. *cruzi* with any major ECG abnormality were at significantly increased risk of death, compared to their counterparts with no such abnormalities, independent of age, sex and other health measures (HR = 1.83; 95% CI 1.44, 2.34). Further adjustments for socioeconomic indicators had little impact on this association (HR = 1.78; 95% CI 1.39, 2.28). The association was consistent across different levels of African, Native American and European genomic ancestries. We found no evidence of statistically significant multiplicative interactions between African, Native American and European genome ancestry levels and major ECG abnormalities on mortality (p>0.05 for all).

**Table 5:** Hazard ratio for 15-year mortality by the presence of any major electrocardiogram abnormalities among infected with *Trypanosoma cruzi* and stratified by individual proportion of African, Native American and European ancestry levels (The Bambui-Epigen Cohort Study of Aging).

ECG abnormality and ancestry level in quintiles	No. Deaths (Death rate per 1,000 pyrs)	HR (95% CI) adjusted for age, sex and health measures^a^	HR (95% CI) adjusted for age, sex, health measures^a^ and socioeconomic indicators^b^
**All**			
No ECG abnormality	100 (38.2)	1.0	1.0
ECG abnormality	97 (74.8)	1.83 (1.44, 2.34)	1.78 (1.39, 2.28)
**African ancestry**			
No ECG abnormality	100 (38.2)	1.0	1.0
ECG abnormality			
Lowest (<4.2%)	20 (59.3)	1.25 (0.78, 2.02)	1.13 (0.70, 1.84)
2nd-4th (4.2%-19.9%)	122 (77.7)	1.93 (1.47, 2.54)	1.90 (1.43, 2.51)
Highest (≥20%)	55 (75.5)	1.75 (1.24, 2.48)	1.77 (1.25, 2.50)
**Native American**			
No ECG abnormality	100 (38.2)	1,0	1.0
ECG abnormality			
Lowest (<2.3%)	23 (64.7)	1.47 (0.96, 2.24)	1.45 (0.93, 2.27)
2nd-4th (2.3%-9.3%)	196 (73.9)	1.78 (1.34, 2.38)	1.74 (1.29, 2.33)
Highest (≥9.4%)	58 (81.8)	1.96 (1.43, 2.68)	1.91 (1.40, 2.62)
**European**			
No ECG abnormality	100 (38.8)	1.0	1.0
ECG abnormality			
Lowest (<71%)	58 (79.9)	1.90 (1.37, 2.63)	1.90 (1.37, 2.63)
2nd-4th (71%-92.7%)	116 (72.0)	1.82 (1.39, 2.39)	1.76 (1.33, 2.32)
Highest (≥93%)	23 (77.2)	1.40 (0.89, 2.22)	1.37 (0.86, 2.17)

ECG, electrocardiogram; pyrs, person-years at risk; HR (95% CI), hazard ratio (95% confidence interval) estimated by Cox proportional hazards regression.

^a^Smoking, body mass index, hypertension, diabetes, coronary heart disease, log high sensitivity C-reactive protein and non-HDL cholesterol plus cluster in family.

^b^Schooling level, monthly household income per capita and father’s occupation.

As shown in Table 6, a statistically significant association between Western African proportion and *T*. *cruzi* infection was found in bivariate analysis, but the association lost significance after adjustments for socio demographic characteristics and health measures. Furthermore, we did not find any evidence of an association between the above mentioned ancestry levels and the presence of major ECG abnormalities among people infected with *T*. *cruzi* in either univariate or multivariate analyses (p>0.05 for both). Finally, as previous observed for global African ancestry, levels of Western African ancestry did not modify the association between a major ECG abnormality and subsequent mortality among infected subjects (p value for interactions >0.05).

**Table 6:** Association between individual proportion of Western African sub-continental genomic ancestry levels relative to total African ancestry with *Trypanosoma cruzi* infection and any major electrocardiogram abnormalities among infected (The Bambui-Epigen Cohort Study of Aging).

Proportion of genomic Western African ancestry^a^ in quintiles	Infected with *T*. *cruzi*		Any major ECG abnormality among infected with *T*. *cruzi*	
	No. (%)	Adjusted PR (95% CI)^b^	No. (%)	Adjusted PR (95% CI)^b^
Lowest (<33.3%)	79 (29.4)	1.0	49 (62.0)	1.0
2^nd^-4^th^ (33.3%-73.3%)	327 (40.6)	0.97 (0.79, 1.19)	187 (57.2)	0.88 (0.70, 1.10)
Highest (≥73.3%)	99 (36.9)	0.99 (0.78, 1.26)	49 (49.5)	0.80 (0.58, 1.09)
	p = 0.004		p = 0.221	

ECG, electrocardiogram; PR (95% CI), prevalence ratio (95% confidence interval) estimated by Poisson regression; p, p value—Pearson´s chi square test.

^a^Relative to total genomic African ancestry.

^b^Age, sex, smoking, body mass index, hypertension, diabetes, coronary heart disease, log high sensitivity C-reactive protein and non HDL cholesterol, schooling level, monthly household income per capita and father’s occupation plus cluster in family.

## Discussion

The key findings of the current study are: first, *T*. *cruzi* infection was strongly correlated with both African and Native American ancestry—and conversely showed a negative correlation with European ancestry—and this association had a graded effect; second, cardiomyopathy in infected persons was not associated with either African or Native American or European ancestry levels; third, genomic ancestry had no significant effect modification on the prognostic value of major ECG abnormalities for mortality in *T*. *cruzi* infected older adults; fourth, Western African sub continental origin was not associated with either *T*. *cruzi* infection or related outcomes. The above-mentioned findings were independent of an array of sociodemographic and biological confounders.

The association between *T*. *cruzi* infection and higher levels of African and Native American ancestry may result from genetic influence on susceptibility and/or greater exposure to infection in these groups during the life course. Our study population was born before 1940, and this cohort has experienced dramatic political and social changes during their lifetimes. Brazil has transitioned from a low-income, primarily rural country in the mid-1950s, to one of the largest economies in the world, with 84% of the population living in urban areas by 2010 [32,33]. Chagas disease is related to poor socio-economic circumstances, mostly in early life. In endemic areas, the main source of infection is a bloodsucking triatomine insect that colonizes poor households. Most individuals in these areas acquire the infection before they reach 20 years of age [34]. Further, ethnoracial disparities in Brazil are remarkable. Persons of African origin are more likely to have lower income and education, to experience race-based discrimination, and to report worse health outcomes [14,35]. Native Americans experience sustained marginalization [36]. Our results are in agreement with these observations, revealing higher levels of African and Native American ancestry in those with lower schooling and family income levels, as well as those whose fathers were rural workers or had an unknown occupation (which suggests a less prestigious occupational category). *T*. *cruzi* infection followed this trend, with higher prevalence associated with worse current (measured by income) and worse early socioeconomic circumstances (educational attainment and father’s occupation). However, the association between higher levels of African and Native American ancestry with *T*. *cruzi* infection was attenuated, but still remained largely significant after adjustments for socioeconomic indicators, suggesting a possible independent effect of genomic ancestry. Despite this finding, it is important to emphasize that although we control for several important measures of current and early socioeconomic circumstances, they cannot completely account for the complexity of unfavorable trajectories of persons with higher levels of African and Native American ancestry in Brazilian society [14]. Thus, we cannot exclude the possibility that residual confounding may still account for the association between higher levels of African and Native American ancestry and prevalent *T*. *cruzi* infection in our analysis. The fact that analyses of subsequent complications (cardiomyopathy) showed no association with genomic ancestry further tempers any inference regarding a causal relationship between genetic ancestry and increased vulnerability to *T*. *cruzi*.

Chronic Chagas cardiomyopathy is the most clinically relevant manifestation of the disease. It manifests as heart failure, arrhythmia, heart block, thromboembolism, stroke and sudden death [7,16]. The pathogenesis of chronic chagasic cardiomyopathy is not completely understood [37], but inflammation caused by persistent parasitism of the heart tissue appears to play an important role [38,39]. Additionally, a recent genome-wide study (GWAS) identified suggestive single nucleotide polymorphisms (SNPs) that may impact the risk of progression to cardiomyopathy in seropositive persons [37]. Electrocardiography has been considered an important tool in the management of ChD patients [7]. Information on ECG findings among the elderly infected with ChD is scant, and very few studies in middle-aged or older adults have used core-lab readings using classifications developed by the internationally accepted Minnesota Code [8]. A previous study in the Bambui cohort showed that any major ECG abnormality (classified by the Minnesota Code) was strongly and independently associated with increased risk for 10-year mortality among *T*. *cruzi* infected older adults [8]. The results of the current analysis, based on an extended 15 year-follow-up, are in agreement with these findings. Additionally, we found no evidence of an association between African and Native American ancestries and major ECG abnormalities among *T*. *cruzi* infected persons. The absence of an association was consistent in bivariate analyses as well as those adjusted for an array of potential confounding factors. Furthermore, African, Native American and European ancestry showed no significant interactions affecting the ability of major ECG abnormalities to predict subsequent mortality.

Strengths of this study include the large population-based cohort followed for an extended period, and minimal loss of participants to follow-up. Another major strength is the use of genome-wide measures of ancestry. Genomic ancestry does not change over time, while ethnoracial self-classification is prone to misclassification—particularly in admixed populations [14,19]. Another strength is the inclusion of several biological and non-biological risk factors in our analysis. However, one cannot exclude the possibility that there may be additional unmeasured factors, including unknown genetic factors that confound our results.

The current study is, to our knowledge, the first investigation on the influence of African, Native American and European genomic ancestry on *T*. *cruzi* infection and related outcomes. Our findings indicate that African and Native American ancestry have no influence on the presence of major ECG abnormalities and had no influence on the ability of an ECG abnormality in predicting mortality in older people infected with *T*. *cruzi*. In contrast, our results revealed a strong positive association between prevalent *T*. *cruzi* infection with higher levels of African and Native American ancestry. Whether this association is a consequence of genetic background, differential exposure to infection, or a combination of both factors, remains to be determined.
